# Comparison of rapid and conventional methods for investigating of *mecA* presence in *Staphylococcus* Species

**DOI:** 10.12669/pjms.37.5.4274

**Published:** 2021

**Authors:** Selim Gorgun, Hacer Isler, Mehmet Cenk Turgut

**Affiliations:** 1 Selim Gorgun, Department of Microbiology and Clinical Microbiology, Health Sciences University, Samsun Training and Research Hospital, Samsun, Turkey; 2 Hacer Isler, Department of Microbiology and Clinical Microbiology, Health Sciences University, Samsun Training and Research Hospital, Samsun, Turkey; 3 Mehmet Cenk Turgut, Department of Orthopedics and Traumatology, Health Sciences University, Regional Training and Research Hospital, Erzurum, Turkey

**Keywords:** *Staphylococcus*, *mecA*, Chromogenic agar, Methicillin Resistance

## Abstract

**Objectives:**

Taking the determination of *mecA* gene by polymerized chain reaction (PCR) method as a reference in determining methicillin resistance in *Staphylococcus* species, we aimed at comparing the reliability levels of disk diffusion, latex agglutination test and chromogenic agar use methods.

**Methods:**

This prospective study was conducted on 228 *Staphylococcus* strains isolated between January 2020 and December 2020 in Samsun Training and Research Hospital. Disk diffusion, latex agglutination and chromogen agar medium methods were applied along with the polymerized chain reaction (PCR) method.

**Results:**

The *mecA* gene was detected in 47 of the isolates (20.6%) by the PCR method, and these isolates were accepted as methicillin-resistant. When the PCR result was taken as a reference, the sensitivity of the disk diffusion method became 100%, and specificity became 45.9%; sensitivity of latex agglutination was determined as 80.9%, and specificity as 70.2%; sensitivity of chromogenic agar as 85.1% and its specificity was found to be 95%. Only in *S. aureus* isolates, the highest sensitivity and specificity rate (100% and 88%, respectively) belonged to chromogenic agar.

**Conclusion:**

Chromogenic agar provides more reliable data for *S. aureus* isolates, and the combined use of all three methods does not significantly increase reliability.

## INTRODUCTION

Although *Staphylococcus* species are a member of the normal skin flora, they can cause a variety of infections ranging from simple wound infections to bacteremia and sepsis. *Staphylococci* are among the most common isolated agents in wound infections. The type of these agents and their resistance to antibiotics are of great importance for determining the treatment of infections.^1^-^3^ Despite the fact that coagulase-negative *Staphylococcus* species cause more frequent infections, *Staphylococcus aureus* is known to be more pathogenic due to its various enzymes and factors.^4^,^5^

Methicillin resistance is the most important factor in the management of infections caused by *Staphylococcus* and the selection of antimicrobials. Methicillin-resistant *Staphylococci* are also resistant to beta-lactam group antibiotics in vivo, which significantly limits antibiotic options.^4^-^6^ In such cases, it is necessary to use antibiotic groups such as carbapenems, which leads to the development of significant resistance in some bacteria such as *Acinetobacter* and *Pseudomonas* that cause hospital infection. In such cases, antibiotics with a high rate of undesirable effects such as vancomycin or teicoplanin can also be an option. However, the development of resistance to this group of antibiotics, which is the most effective against *Staphylococcus*, constitutes one of the most dreadful scenarios in the world.^4^-^8^

Correct determination of the antibiotic resistance profile of *Staphylococcus* isolates obtained from the infection is critical for the management of the infection. Different methods have been tested for a long time to accurately determine whether there is methicillin resistance or not. Although molecular methods used for the detection of *mecA* and *mecC* genes that cause methicillin resistance are accepted as reference methods, for now, these methods cannot be used in routine applications. For this reason, the reliable detection of methicillin resistance by conventional methods has become a preferred necessity in terms of being both cost-effective and easy to apply.^4^,^7^,^9^,^10^

In this study, we aimed at comparing the reliability levels of disk diffusion, latex agglutination test, and chromogenic agar methods, by taking the *mecA* gene detection by polymerized chain reaction (PCR) as a reference in determining methicillin resistance in *Staphylococcus* species.

## METHODS

This study has been approved by the local ethics committee on 2020/13/8 and planned prospectively.

### Obtaining and Identifying Strains

A total of 228 staphylococcal isolates obtained from various clinical samples sent to Samsun Training and Research Hospital’s microbiology laboratory between January 2020 and December 2020 were included in the study. In the laboratory colony morphology, pigment formation, Gram’s staining, catalase, and coagulase assays were concluded on the clinical specimens. Bacteriel identifications were detected by Vitec 2 (bioMérieux, France).

In the study, *S. aureus* ATCC 4103, *S. aureus* ATCC 25923, *S. saprophyticus* ATCC 45678, *S. xylosus* ATCC 95055, *S. hominis* ATCC 51624, *S. capitis* ATCC 56789, *S. epidermidis* ATCC 10003 were used as control strains.

Considering that it may belong to the same origin, only one of the isolates obtained from the same patient was included in the study. Similarly, only one of the isolates obtained from patients hospitalized in the same service (e.g. intensive care unit) on the same day was included in the study. Isolates that were not considered growth was far below the amount of a causative pathogen were excluded from the study.

### Determining Methicillin Resistance:

### Disc Diffusion Method:

Cefoxitin susceptibility of *Staphylococcus* strains was determined by disc diffusion method. Discs containing 30 μg Cefoxitin (Oxoid, Ireland) were used. For each strain, the solutions prepared with 0.5 McFarland turbidity were seeded on Mueller Hinton agar and the methicillin sensitivity was evaluated after 24 hours of incubation.^11^

### Latex Agglutination

The presence of penicillin-binding protein 2a (PBP2a) in the isolates was determined using the Slidex MRSA kit (bioMérieux, France) and the test was carried out according to the manufacturer’s recommendations. Positive control was used for each test.

### Chromogenic Agar

In the study, chromID® MRSA SMART kit (bioMérieux, France) was used, as well as a chromogenic medium used for screening methicillin resistance. The microorganism was cultivated on chromogenic agar under aseptic conditions. It required 18-24 hours of incubation. Methicillin-sensitive *S. aureus* (MSSA) could not grow on chromogenic agar, while methicillin-resistant *S. aureus* (MRSA) grown in a pinkish color. Other bacteria either could not reproduce or grow blue or colorless.^12^,^13^

### MecA PCR:

The multiplex PCR protocol used in the study was as follows: 10x PCR Buffer 2.5µL, 10 mM dNTP 0.5 µL, *MECA* 1 (10 pmol) 1.25 µL, *MECA* 2 (10 pmol) 1.25 µL, 25 mM MgCl23 µL, DNA Polymerase 0.5 µL, distilled water 13.5 µL and bacterial DNA 2.5 µL. Thermal Cycler phase: 1 cycle at 94^o^C is 2 minutes, at 94^o^C 35 cycles 15 sec, at 55^o^C 35 cycles 30 seconds, 72^o^C 35 cycles 30 seconds and 72^o^C 1 cycle 10 minutes.

### Statistical analysis

All statistical analyzes in the study were performed using SPSS 25.0 software (IBM SPSS, Chicago, IL, USA). Descriptive data were given in numbers and percentages.

Sensitivity, specificity, positive predictive value, negative predictive value, and accuracy of the methods were calculated. For sensitivity, the ratio of the number of isolates found to be resistant to methicillin to the actual number of resistant isolates, and for specificity, the ratio of the number of isolates found to be susceptible to methicillin to the actual number of susceptible isolates was calculated. The positive predictive value was calculated from the ratio of the true resistant isolates among the isolates found to be resistant to methicillin, and the negative predictive value from the ratio of the actual susceptible isolates among the isolates found to be susceptible to methicillin. Finally, accuracy was calculated as the ratio of the total number of methicillin-resistant and susceptible isolates determined by the method to the total number of isolates.

## RESULTS

In the study, 45.6% of the isolates were obtained from blood, 41.2% from wounded tissues, 11.4% from tracheal aspirate cultures, and 1.8% from cerebrospinal fluid. Of the isolates 34 (14.9%) were *S. aureus*, 28 (12.3%) were *S. epidermidis*, 23 (10.1%) were *S. hominis*, 21 (9.2%) were *S. capitis*, 20 (8.8%) were *S. haemolyticus*, 19 (8.3%) were *S. warneri* and 83 (36.4%) were other *Staphylococcus* species.

*MecA* gene was detected in 47 (20.6%) of the isolates by using PCR method and these isolates were accepted as methicillin-resistant (Fig. 2). 145 (63.6%) isolates were found to be methicillin-resistant by disk diffusion method, 92 (40.4%) were found resistant by latex agglutination, and 49 (21.5%) were found resistant by chromogenic agar (Table-I).

**Table-I:** Performance of three methods according to PCR result in all isolates.

	PCR		Total	Sensitivity (%)	Specificity (%)	PPV (%)	NPV (%)	Accuracy (%)

	R (n=47)	S (n=18)						
Disc diffusion				100	45.9	32.4	100	57.0
R	47	98	145					
S	0	83	83					
Latex agglutination				80.9	70.2	41.3	93.4	72.4
R	38	54	92					
S	9	127	136					
Chromogenic agar				85.1	95.0	81.6	96.1	93.0
R	40	9	49					
S	7	172	179					

PCR: Polymerized chain reaction, PPV: Positive predictive value, NPV: Negative predictive value, S: Methicillin sensitive, R: Methicillin resistant.

**Fig.1:**
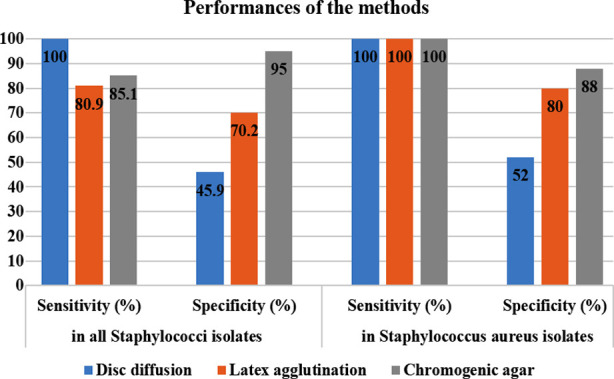
Sensitivity and specificity values of the methods.

Only *S. aureus* isolates had the highest sensitivity and specificity rate for chromogenic agar (100% and 88%, respectively). The sensitivity of the chromogenic agar method in coagulase-negative *Staphylococcus* was 81.6% and its specificity was determined as 96.2% (Table-II) (Fig.1).

**Table-II:** Performance of three methods according to PCR result in Staphylococcus aureus isolates.

	PCR		Total	Sensitivity (%)	Specificity (%)	PPV (%)	NPV (%)	Accuracy (%)

	R (n=9)	S (n=25)						
Disc diffusion				100	52	42.9	100	64.7
R	9	12	21					
S	0	13	13					
Latex agglutination				100	80	64.3	100	85.3
R	9	5	14					
S	0	20	20					
Chromogenic agar				100	88	75	100	91.2
R	9	3	12					
S	0	22	22					

PCR: Polymerized chain reaction, PPV: Positive predictive value, NPV: Negative predictive value, S: Methicillin sensitive, R: Methicillin-resistant.

**Fig.2:**
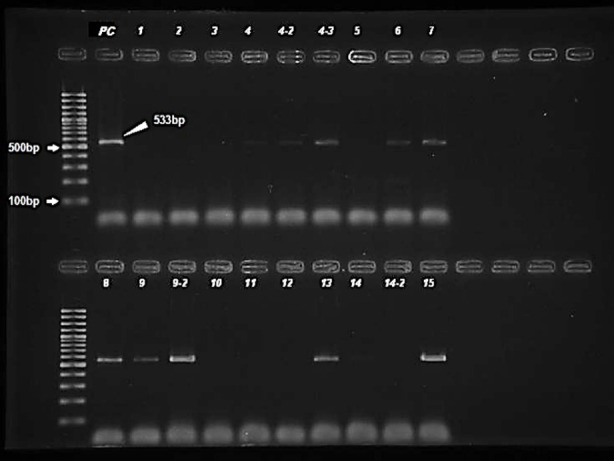
PCR amplification of *MecA* gene. Expected aplicon size is 533 bp. M 100 bp DNA ladder was used. Lane 1 (PC) was the positive control.

The sensitivity and specificity of the chromogenic agar method were 100% and 88% for *S. aureus*, respectively; 100% and 100% for *S. epidermidis* and *S. sciuri* and 100% and 94.7% for *S. hominis*. The sensitivity and specificity of the chromogenic agar method were 100% and 88% for *S. aureus*, respectively; 100% and 100% for *S. epidermidis* and *S. sciuri* and 100% and 94.7% for *S. hominis*. The sensitivity and specificity of the latex agglutination method were determined as 100% and 94.4% for *S. capitis*, 100% and 89.5% for *S. hominis*. While most of the sensitivities of all three methods were found to be 100% for *Staphylococcus* species, specificity rates were observed to vary greatly. Among these common species, the median sensitivity of all three methods was 100%. The specificity rates of disc diffusion were between 14.3% and 61.1% (median: 47.4% [interquartile range; IQR: 16.3%]), and latex agglutination were between 50% and 94.4% (median: 76.9% [IQR: 28.0%]), and chromogenic agar were between 75% and 100% (median: 100% [IQR: 12.0%]). (Table-III).

**Table-III:** Performance comparison of methods based on *Staphylococcus* species.

	n	Disc diffusion		Latex agglutination		Chromogenic agar	

		Sensitivity (%)	Specificity (%)	Sensitivity (%)	Specificity (%)	Sensitivity (%)	Specificity (%)
S.aureus	34	100	52	100	80.0	100	88.0
S.epidermidis	28	100	53.8	100	76.9	100	100
S.haemolyticus	20	100	38.5	100	61.5	57.1	100
S.capitis	21	100	61.1	100	94.4	33.3	100
S.sciuri	19	100	14.3	100	50.0	100	100
S.hominis	23	100	47.4	100	89.5	100	94.7
S.saprophyticus	13	100	37.5	0,0	75.0	100	75.0
Median		100	47.4	100	76.9	100	100
Interquartile range		0	16.3	0	28.0	42.9	12.0

Analysis was made considering the combination of methods, and the isolate detected by any method as susceptible to methicillin was considered susceptible. According to the analysis results, it was found that the combination of disk diffusion and chromogenic agar had the best performance with a sensitivity of 85.1% and its specificity was 95% (Table-IV). When the isolate found to be resistant to methicillin by any method is accepted as resistant; The sensitivity of the combination of latex agglutination and chromogenic agar, with the best performance, was 100% and its specificity was 68.5% (Table-V).

**Table-IV:** Isolates determined to be susceptible by any method are considered susceptible, performance of combinations of three methods according to PCR result in all isolates.

	PCR		Total	Sensitivity (%)	Specificity (%)	PPV (%)	NPV (%)	Accuracy (%)

	R (n=47)	S (n=181)						
DD + LA				80.9	81,2	52.8	94.2	81.1
R (Both methods)	38	34	72					
S (Any single method)	9	147	156					
DD + CA				85.1	95	81.6	96.1	93
R (Both methods)	40	9	49					
S (Any single method)	7	172	179					
LA + CA				66	96.7	83.8	91.6	90.4
R (Both methods)	31	6	37					
S (Any single method)	16	175	191					
DD + LA + CA				66	96.7	83.8	91.6	90.4
R (Both methods)	31	6	37					
S (Any single method)	16	175	191					

Isolates determined to be "susceptible" by any method are considered susceptible. PCR: Polymerized chain reaction, PPV: Positive predictive value, NPV: Negative predictive value, S: Methicillin sensitive, R: Methicillin-resistant, DD: Disc diffusion, LA: Latex agglutination, CA: Chromogenic agar.

**Table-V:** Isolates determined to be resistant by any method are considered resistant, performance of combinations of three methods according to PCR result in all isolates.

	PCR		Total	Sensitivity (%)	Specificity (%)	PPV (%)	NPV (%)	Accuracy (%)

	R (n=47)	S (n=181)						
DD + LA				100	34.8	28.5	100	48.2
R (Any single method)	47	118	165					
S (Both methods)	0	63	63					
DD + CA				100	45.9	32.4	100	57
R (Any single method)	47	98	145					
S (Both methods)	0	83	83					
LA + CA				100	68.5	45.2	100	75
R (Any single method)	47	57	104					
S (Both methods)	0	124	124					
DD + LA + CA				100	34.8	28.5	100	48.2
R (Any single method)	47	118	165					
S (Both methods)	0	63	63					

Isolates determined to be "resistant" by any method are considered resistant. PCR: Polymerized chain reaction, PPV: Positive predictive value, NPV: Negative predictive value, S: Methicillin sensitive, R: Methicillin-resistant, DD: Disc diffusion, LA: Latex agglutination, CA: Chromogenic agar.

## DISCUSSION

*Staphylococcus* is one of the bacteria isolated as the most common infectious agent and wound infection pathogen (between 19% and 58.5%).^14^-^17^ The presence of methicillin resistance is one of the most important guiding factors in the treatment of staphylococcal infections. Although some molecular methods and several dilution methods (for determining the minimum inhibitory concentration (MIC) value) have been developed to determine methicillin resistance in Staphylococcus species, they are not ideal for use in routine laboratory applications.^4^-^7^ Automated systems also determine methicillin resistance by determining the MIC value, but in many small-scale laboratories, these systems are not included for not being cost-effective. For these reasons, we focused on detecting methicillin resistance with easy and fast methods that can be applied in every laboratory. However, there remain uncertainties about whether these methods provide reliable data.^4^,^7^,^8^ The performance of three different methods was evaluated in our study.

One of the most classical methods for determining methicillin resistance in *Staphylococcus* is the disk diffusion method. Oxacillin has been used for a long time for this purpose. However, after it was determined that the reliability level was much lower than expected, oxacillin was replaced by cefoxitin, which gave better results. It has been reported that cefoxitin expresses the *mecA* better than other penicillins.^18^ However, the reliability of the disk diffusion method has been remaining under questioning so far. In some studies, it has been reported that the sensitivity of the disk diffusion test using cefoxitin disc is between 94% and 100% and the specificity is between 96% and 100% for determining methicillin resistance in *Staphylococcus*.^19^-^24^ When the PCR result is taken as a reference in this study, the sensitivity of the disk diffusion method in all *Staphylococcus* isolates was 100% and specificity was determined to be 45.9%. Considering the highest number of isolated *Staphylococcus* in the study, the sensitivity of the disk diffusion method was 100% for all the isolates; also it was determined that the specificity rates varied between 14.3% and 61.1% (median: 47.4% [IQR: 16.3%]). Accordingly, it was observed that the negative predictive value of the disk diffusion method was 100%, but the positive predictive values were below 50%. All these findings show that in *Staphylococcus*, the disk diffusion method, in general, captures methicillin resistance in almost all isolates. However, it is seen that the specificity rates for methicillin resistance are far below the acceptable level; that is, false-positive results are very high for methicillin resistance. This may cause the patient to use strong antibiotics unnecessarily when a methicillin-sensitive *Staphylococcus* is isolated as the agent. In the light of this, the disk diffusion method seems to be far from providing reliable data in both *S. aureus* isolates and coagulase-negative *Staphylococcus*.

MRSA isolates produce PBP2a, which has a lower affinity for beta-lactam antibiotics than PBP2. The *mecA* encodes PBP2a, which is the target of methicillin.^12^,^20^ The latex agglutination test used in our study is a serological method used for *S. aureus* isolates, determining the presence of PBP2a, and can be applied easily and quickly. In some studies, it has been reported that the sensitivity of the latex agglutination method using cefoxitin disc is between 88% and 100%, and specificity is between 97% and 100% in determining methicillin resistance in *Staphylococcus*.^12^,^20^-^22^,^25^ In our study, the sensitivity of the latex agglutination method was found to be 80.9% and specificity was found to be 70.2% in all *Staphylococcus* isolates. Only in *S. aureus* isolates, the sensitivity and specificity rates were 100% and 80%, respectively, and 76.3% and 68.6% for general coagulase-negative *Staphylococcus*, respectively. In most of the *Staphylococcus* species detected in high numbers in our study, the sensitivity of this method was determined as 100%, whereas the specificity rates dispersed between 50% and 95%. Specificity rates were found to be high in *S. sciuri* and *S. hominis* isolates, but methicillin resistance could not be determined in any of the *S. saprophyticus* isolates. These findings show that the latex agglutination method has a high sensitivity rate in determining methicillin resistance in general, its specificity for *S. aureus* is below the expected, and it is not usable in coagulase-negative *Staphylococcus* due to its highly unsteady performance.

The chromogenic agar used in our study is a medium that prevents the growth of methicillin-resistant *Staphylococcus*, because of the cefoxitin it contains. In addition, it indicates whether the growing colony is *S. aureus* or not based on the enzymes it contains. In this medium, the main purpose is to detect MRSA.^12^,^13^,^26^ In some studies, it has been reported that the sensitivity of the disk diffusion test using cefoxitin disc in determining methicillin resistance in *Staphylococcus* is between 75% and 100% and the specificity is between 89% and 100%.^23^,^27^-^31^ The sensitivity of chromogenic agar in our study was 85.1% and its specificity was determined as 95%. The sensitivity and specificity rates were 100% and 88%, respectively, in *S. aureus* isolates only; 81.6% and 96.2% in coagulase-negative *Staphylococcus*. Sensitivity and specificity rates were found to be 100% in some *Staphylococcus* species. Among these common species, the sensitivity [IQR: 42.9%] and specificity [IQR: 12.0%] median values of chromogenic agar were found to be 100%. It was observed that the highest sensitivity and specificity rate for *S. aureus* isolates belonged to chromogenic agar among three methods. All these findings show that the use of chromogenic agar for the determination of methicillin in *S. aureus* isolates has yielded significantly reliable results, but its specificity is still somewhat low. According to these data, the reliability level of chromogenic agar generally gives relatively high results except for *S. aureus*.

In our study, in case the methods are used in combination, two separate analyzes were carried out. When the isolate determined by any method as susceptible to methicillin was considered sensitive, it was observed that the combination of disk diffusion and chromogenic agar showed the best performance with a sensitivity rate of 85.1% and the specificity rate of 95%.

In cases where the methods were performed in combination, two analyses were carried out. When the isolate determined as resistant to methicillin by any method was considered as resistant, it was determined that the combination of latex agglutination and chromogenic agar showed the best performance, and the sensitivity was 100% and the specificity was 68.5%. These findings show that the combined use of these methods either increases sensitivity or specificity but cannot increase both sensitivity and specificity. Accordingly, besides not providing high-reliability data, it is seen that their combined use does not significantly increase the level of reliability.

### Limitations of the study

Since the study aimed at comparing routinely-used methicillin resistance detection methods, determination of minimum inhibitory concentration (MIC) values with seldomly used micro and macro dilution methods were not included. In addition, the presence of *mecC* genes was not investigated in the study. Methicillin resistance depends on the presence of the *mecA* or *mecC* genes, but the prevalence of *mecC* gene presence has been reported to be 0.009%^32^, and it was deemed that our study would not significantly affect the analysis results.

## CONCLUSION

The data of our study show that disk diffusion, latex agglutination, and chromogenic agar methods do not yield high-reliability results in determining methicillin resistance in *Staphylococcus* species, but chromogenic agar provides more reliable data in *S. aureus* isolates, and the combined use of all three methods does not significantly increase the reliability level.

### Author Contribution:

**SG, HI, MCT:** Conceptualization and design of study.

**SG, HI:** Methodology, takes the responsibility for integrity of the study.

**SG, HI, MCT:** Data processing. SS, MCT: Statistical Analyse.

**SG, HI, MCT:** Writing-editing.
